# Is adjuvant radiotherapy warranted in resected pT1-2 node-positive rectal cancer?

**DOI:** 10.1186/1748-717X-8-290

**Published:** 2013-12-19

**Authors:** Junjie Peng, Xinxiang Li, Ying Ding, Debing Shi, Hongbin Wu, Sanjun Cai

**Affiliations:** 1 Department of Colorectal Surgery, Fudan University Shanghai Cancer Center, Shanghai, China; 2 Department of Oncology, Shanghai Medical College, Fudan University, Shanghai, China; 3 Department of Biostatistics, University of Pittsburgh, Pittsburgh, PA, USA

**Keywords:** Rectal cancer, Radiotherapy, Local recurrence, Stage, Prognosis

## Abstract

**Background:**

Stage T1-2 rectal cancers are unlikely to have lymph node metastases and neoadjuvant therapy is not routinely administered. Postoperative management is controversial if lymph node metastases are detected in the resected specimen. We studied the outcomes of patients with pT1-2 node-positive rectal cancer in order to determine whether adjuvant radiotherapy was beneficial.

**Methods:**

We conducted a retrospective analysis of 284 patients with pathological T1-2 node-positive rectal cancer from a single institution. Outcomes, including local recurrence (LR), distant metastasis (DM), disease free survival (DFS) and overall survival (OS), were studied in patients with detailed TN staging and different adjuvant treatment modalities.

**Results:**

The overall 5-year LR, DM, DFS and OS rates for all patients were 12.5%, 32.9%, 36.4% and 76.8%, respectively. Local control was inferior among patients who received no adjuvant therapy. Patients could be divided into three risk subsets: Low-risk, T1N1; Intermediate-risk, T2N1 and T1N2; and High-risk, T2N2. The 5-year LR rates were 5.3%, 9.8% and 26.4%, respectively (p = 0.005). In High-risk patients, addition of radiotherapy achieved a 5-year LR rate of 9.1%, compared 34.8% without radiotherapy.

**Conclusions:**

In our study, we provide the detailed outcomes and preliminary survival analysis in a relatively infrequent subset of rectal cancer. Three risk subsets could be identified based on local control for pT1-2 node positive rectal cancer. Postoperative treatment needs to be individualized for patients with pT1-2 node-positive rectal cancer.

## Background

Neoadjuvant chemoradiotherapy (CRT) is recommended for clinical T3N0 and node-positive rectal cancers based on studies demonstrating a decrease in local recurrences and associated morbidity [1-3]. When confined within the muscularis propria layer (T1-2), rectal cancers are less likely to have spread to regional lymph nodes and a considerable proportion of patients will receive primary surgery. Adjuvant treatment is appropriate when pathological lymph nodes are detected in the resected specimen of T1-2 tumors. Outcomes data for this small subset of patients with pT1-2 node-positive rectal cancer mainly derives from pooled analyses, in which the local control rate was not adequately addressed [4,5]. Therefore, the optimal adjuvant treatment for patients with T1-2 node-positive rectal cancer is still in question.

Our series, a retrospective study from a single institution, was designed to assess the outcomes of patients with pT1-2 node-positive rectal cancer who underwent primary surgery followed by adjuvant chemotherapy, CRT, or no adjuvant therapy. Our principal objective was to determine the impact of adjuvant radiotherapy in subsets of patients with pT1-2 node-positive rectal cancers.

## Materials and methods

### Patients and treatment modalities

This study was approved by the Fudan University Shanghai Cancer Center Institutional Ethics Committee, and written informed consent was obtained from the patient for the publication of this report and any accompanying images. 284 patients with pathological T1-2 (pT1-2) node-positive rectal cancer were treated and included in an institutional database at Fudan University Shanghai Cancer Center between January 1993 and December 2009. The primary site was considered to be in the rectum if the tumor was located within 15 cm from the anal verge by digital examination or endoscopy. In this study, the 6th edition of the American Joint Committee on Cancer’s (AJCC) Cancer Staging Manual was used; [6] we were unable to define N1c patients according to the AJCC 7th edition [7]. Based on an institutional follow-up protocol, patients were evaluated every 6 months for the first three years after surgery, and each year thereafter. Serum carcinoembryonic antigen (CEA), chest X-ray/computed tomography scanning, and abdomen and pelvic ultrasound/CT scanning was typically performed according to surgeons’ guidance.

Between September and December 2012, all surviving patients were contacted by telephone or mail in addition to their scheduled follow up to update records for the database. Sixty-five cases were excluded: 56 cases (19.7%) were lost to follow-up; 9 cases (3.2%) had received neoadjuvant chemotherapy. Therefore, a total of 219 patients (77.1%) with AJCC pT1-2 node-positive rectal cancers were included in this analysis.

The patterns of care at the Fudan University prior to 2006 did not include pelvic MRI as a preoperative staging technique or neoadjuvant CRT for middle and low rectal cancers. Before 2006, patients with cT1-2 disease rarely received preoperative treatment regardless of node status. All patients in this study underwent primary total mesorectal excision (TME) together with either abdominoperineal resection (APR) or anterior resection (AR) of mid and low rectal cancers by colorectal surgeons. As a retrospective study, the regimens of adjuvant chemotherapy varied, including 5-fluorouracil (5-Fu) based monotherapy (5-Fu/leucovorin or capecitabine) and combined chemotherapy (FOLFOX4, mFOLFOX6 and XELOX regimens). For patients receiving adjuvant CRT, 5-fluorouracil based chemotherapy was delivered concurrently with radiation therapy. According to institutional routine, post-operative radiotherapy is the North American standard of care [8]. The total dose of radiation in our series was 45-55 Gy. The standard fractionation is 45 Gy in 25 fractions to the pelvis in 5 weeks with an optional reduced volume boost of 5.4–9 Gy in 3–5 fractions to tumor bed. There were two phases based on radiation technique application. Four fields with block were used before 2002 and 3D conformal therapy was applied afterwards.

### Statistics

Local recurrence (LR) is defined as recurrent tumor at the anastomosis, pelvic viscera, parietal pelvic structures and/or presacral or sacral bone invasion, determined by physical examination, endoscopy or imaging. Distant Metastases (DM) is defined as metastases at distant organs or structures (lymph nodes, etc.). The rate of LR or DM is the cumulative actuarial incidence of local recurrence or distant metastases. Disease-free survival is defined as the time to local recurrence, or distant metastases, or death (whichever occurs first). For each event (LR, DM and death), the time to event was calculated from the completion of surgery to the occurrence of that event. The rates of LR, DM, disease-free survival (DFS) and OS were computed using the Kaplan–Meier method. Log-rank tests were performed to compare differences among survival curves in univariate analyses. The distributions of clinicopathological characteristics among different treatment modalities were assessed by Pearson chi-square tests in crosstab tables. The Cox regression model was used in multivariate analyses, and hazard ratios were estimated with corresponding 95% confidence intervals (95% CI). A p-value of < 0.05 was considered statistically significant.

## Results

### Outcomes and prognostic factors

**Figure 1:**
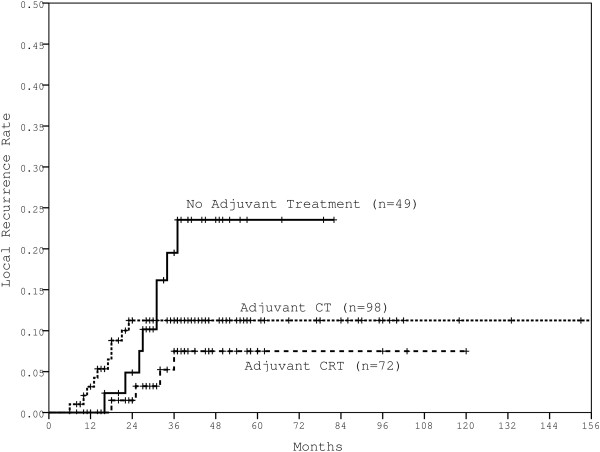
The LR rates in all patients with different adjuvant treatment modalities.

With a median follow-up of 38 months (range, 6–204 months), the 5-year LR, DM, DFS and OS rates for all patients were 12.5%, 32.9%, 63.6% and 76.8%, respectively. Detailed clinicopathological characteristics and treatment are listed in Table 1. Patients who received no adjuvant treatment experienced the highest 5-year LR rate (23.5%); similar local control was observed following adjuvant CT or CRT for all the patients as a whole (LR rates of 11.2% and 7.5%, respectively) (Figure 1). Patients’ clinicopathological characteristics and postoperative treatment were included in univariate analyses comparing different outcomes at 5 years after surgery. Type of surgery was related to 5-year LR rate. N stage was the only variable that was significantly associated with all patients’ outcomes: 5-year LR, DM, DFS and OS rates (Table 1). Although numerical differences were observed in the univariate analyses, patients who received adjuvant CRT did not show statistically improved local control compared to patients who received CT only (5-year LR, 7.5% with CRT vs. 11.2% with CT, p = 0.234).

**Table 1:** Demographic characteristics and different outcomes of patients with pT1-2 node-positive rectal cancer

**Characteristics**		**No. (%) n = 219**	**5 y LR rate(%)**	**P value**	**5 y DM rate(%)**	**P value**	**5 y DFS rate(%)**	**P value**	**5 y OS rate(%)**	**P value**
Gender										
	Male	116 (53.0)	16.6	0.095	38.4	0.305	57.6	0.162	72.4	0.428
	Female	103 (47.0)	8.1		28.5		69.1		81.0	
Age										
	<60	130 (59.4)	13.6	0.618	26.7	0.754	70.4	0.589	81.9	0.378
	≥60	89 (40.6)	10.7		42.8		53.5		70.2	
Tumor Location*										
	Low	155 (70.8)	15.2	0.072	34.1	0.495	61.9	0.976	74.8	0.919
	High-Medium	64 (29.2)	6.1		31.5		66.8		80.6	
CEA(ng/ul)										
	<5	147 (67.1)	12.9	0.964	33.3	0.784	63.5	0.828	76.0	0.384
	≥5	72 (32.9)	12.0		34.7		61.8		74.6	
Tumor Size										
	<3.5	100 (45.7)	13.0	0.935	30.3	0.346	66.8	0.423	73.7	0.898
	≥3.5	119 (54.3)	12.1		35.6		60.8		77.8	
Surgery**										
	AR	114 (52.1)	4.8	0.002	29.9	0.423	69.0	0.621	81.3	0.782
	APR	105 (47.9)	19.9		34.2		59.6		73.3	
Histopathology										
	Adenocarcinoma	200 (91.3)	11.9	0.510	32.6	0.931	64.2	0.522	76.9	0.681
	Mucinous Cancer	19 (8.7)	17.1		33.7		59.9		75.1	
Tumor Grade										
	High	30 (13.7)	12.9	0.978	42.6	0.684	54.5	0.856	67.9	0.912
	Low-Medium	189 (86.3)	12.5		31.8		64.5		77.7	
T Stage										
	T1	61 (27.9)	6.9	0.202	43.1	0.145	57.8	0.746	81.1	0.832
	T2	158 (72.1)	14.3		29.9		64.9		76.1	
N Stage										
	N1	162 (74.0)	8.7	0.007	27.6	0.012	70.4	0.001	81.8	0.002
	N2	57 (26.0)	23.0		49.9		43.9		62.5	
Sampled Lymph Nodes										
	<12	106 (48.4)	9.8	0.291	35.2	0.509	61.8	0.927	77.5	0.864
	≥12	113 (51.6)	15.5		31.0		64.9		76.3	
PNI/LVI										
	No	148 (67.6)	9.7	0.144	28.9	0.028	68.1	0.043	78.3	0.705
	Yes	71 (32.4)	18.7		41.6		53.7		73.2	
Adjuvant Treatment										
	No	49 (22.4)	23.5	0.131	49.5	0.384	48.2	0.930	82.1	0.685
	Adjuvant CT	98 (44.7)	11.2		25.8		69.1		80.5	
	Adjuvant CRT	72 (32.9)	7.5		42.4		56.5		61.3	

*Tumor location: Low, within 6 cm from anal verge; High-Medium, from 6 to 15 cm from anal verge.

**Abbreviations: AR Anterior Resection, *APR* Abdominoperineal Resection, *CT* Chemotherapy, *CRT* Chemoradiotherapy, *PNI* perineural invasion, *LVI* lymphovascular invasion.

Multivariate analyses revealed that N stage (N2 vs. N1) was the only independent prognostic factor for 5-year DM, DFS and OS rates, with hazard ratios of 2.12 (95% CI 1.17-3.83, p = 0.013), 2.37 (95% CI 1.40-4.00, p = 0.001) and 2.89 (95% CI 1.44-5.81, p = 0.003), respectively. N stage (N2 vs. N1) and type of surgery (APR vs. AR) were independent prognostic factors for 5-year LR rate, with hazard ratios of 3.59 (95% CI 1.52-8.48, p = 0.004) and 5.05 (95% CI 1.67-15.28, p = 0.004), respectively. In addition, patients without any adjuvant treatment were at significantly higher risk for local failure, compared with patients with adjuvant CT (HR, 2.98; p = 0.07) or CRT (HR, 5.88; p = 0.007).

### Interaction of treatment modalities and outcomes

Further analyses were performed to assess the importance of different adjuvant treatments for patients’ outcomes. In our series, more patients with high-grade tumors, perineural or lymphovascular invasion received adjuvant CRT (Table 2). The distributions of adjuvant treatments were comparable among different N stage, T stage and type of surgery. The interaction of treatment modalities and patients’ outcomes was presented in Table 3. No statistical significance was found by comparing outcomes in patients with no adjuvant treatment vs. any adjuvant treatment, no adjuvant CRT vs. adjuvant CRT, and no adjuvant treatment vs. adjuvant chemotherapy only.

**Table 2:** The distribution of main clinicopathological characteristics among different treatment modalities

**Treatment information**		**No adjuvant (%) n = 49**	**Adjuvant CT (%) n = 98**	**Adjuvant CRT (%) n = 72**	**P value**
Tumor Size					
	<3.5	22 (10.0)	42 (19.2)	36 (16.4)	0.65
	≥3.5	27 (12.3)	56 (25.6)	36 (16.4)	
Surgery					
	AR	29 (13.2)	49 (22.4)	36 (16.4)	0.53
	APR	20 (9.1)	49 (22.4)	36 (16.4)	
Histopathology					
	Adenocarcinoma	44 (20.1)	90 (41.1)	66 (30.1)	0.91
	Mucinous Cancer	5 (2.3)	8 (3.7)	6 (2.7)	
Tumor Grade					
	High	6 (2.7)	7 (3.2)	17 (7.8)	0.008
	Low-Medium	43 (19.6)	91 (41.6)	55 (25.1)	
T Stage					
	T1	10 (4.6)	28 (12.8)	23 (10.5)	0.37
	T2	39 (17.8)	70 (32.0)	49 (22.4)	
N Stage					
	N1	35 (16.0)	78 (35.6)	49 (22.4)	0.21
	N2	14 (6.4)	20 (9.1)	23 (10.5)	
PNI/LVI					
	No	33 (15.1)	76 (34.7)	39 (17.8)	0.006
	Yes	16 (7.3)	22 (10.0)	33 (15.1)	

**Table 3:** The outcomes and interaction of patients with different treatment modalities

	**5 y LR rate**	**5 y DM rate**	**5 y DFS rate**	**5 y OS rate**
No adjuvant treatment vs. Any adjuvant Treatment (n = 219)				
No Adjuvant Treatment (n = 49)	23.5*	49.5	48.2	82.1
Any Adjuvant Treatment (n = 170)	9.5	31	65.6	75.5
No adjuvant CRT vs. Adjuvant CRT (n = 219)				
No adjuvant CRT (n = 147)	15	29.1	65.9	81.3
Adjuvant CRT (n = 72)	7.5	43.4	56.5	61.3
No adjuvant CT vs. Adjvuant CT only (n = 147)				
No adjuvant CT (n = 49)	23.5	49.5	48.2	82.1
Adjuvant CT only (n = 98)	11.2	25.8	69.1	80.5

*None of the outcomes in this table had statistical significance.

**Figure 2:**
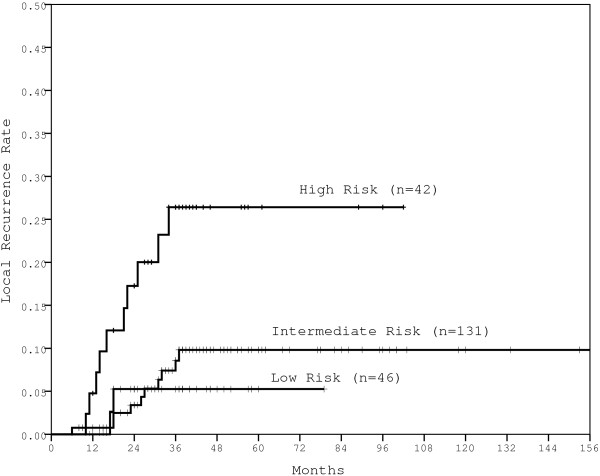
The LR rates for patients within different risk groups.

By re-categorizing patients by more detailed TN stage, significantly different outcomes (5-year LR, DM, DFS and OS rates) were observed among patients with T1N1, T2N1, T1N2 and T2N2 disease (Table 4). Therefore, in our series, patients with T1-2 node-positive rectal cancer could be divided into three risk subsets with significantly different LR rates: Low-risk, T1N1; Intermediate-risk, T2N1 and T1N2; and High-risk, T2N2 (p = 0.005, Figure 2). In Low-risk patients, the 5-year LR rate was only 5.3%, while the 5-year DM rate was 22.9%. In High-risk patients, both the 5-year LR rate and DM rates were as high at 26.4% and 50.6%, respectively, with a significantly lower 5-year OS rate (59.3%, vs. 82.5% for Low-risk).

**Table 4:** Outcomes of patients with rectal cancer in detailed TN stage

**Detailed TN stage**	**T1N1**	**T2N1**	**T1N2**	**T2N2**	**P value**
	**n = 46**	**n = 15**	**n = 116**	**n = 42**	
5 y LR rate (%)	5.3	9.7	12.5	26.4	0.014
5 y DM rate (%)	22.9	42.3	38.1	50.6	0.025
5 y DFS rate (%)	73.8	58.8	61.9	41.0	0.004
5 y OS rate (%)	82.5	82.2	77.1	59.3	0.011

**Figure 3:**
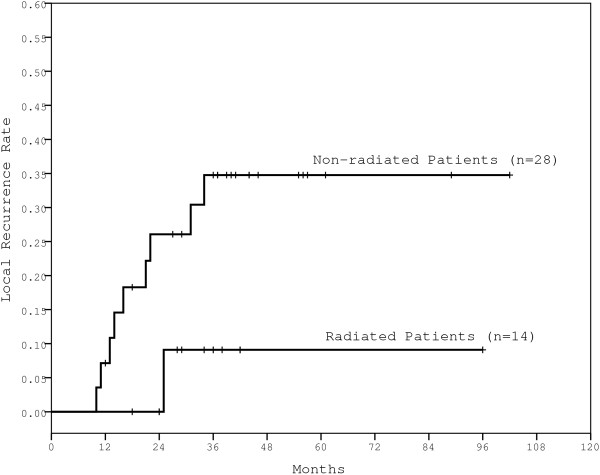
The LR rates for high-risk patients with or without postoperative radiation.

Among the 42 High-risk patients, 14 patients (33.3%) received adjuvant radiotherapy, while the other 28 patients received no adjuvant treatment or adjuvant chemotherapy alone. Only one of the 14 radiated patients experienced a local recurrence for a 5-year LR rate of 9.1%, while 9 of the 28 non-radiated patients experience local recurrence for a 5-year LR rate of 34.8% (Figure 3). A trend was observed favoring adjuvant radiation for patients with T2N2 rectal cancer (p = 0.08).

## Discussion

The management of rectal cancer requires a multidisciplinary team approach and individualized treatment based on assessment of tumor stage, location and surgical resectability. Although preoperative locoregional staging is standard for all patients with rectal cancer, the currently accepted methods, pelvic MRI and EUS, are imperfect, particularly for lymph node staging [9-13]. Retrospective studies have demonstrated a relatively lower risk of lymph node metastasis in T1-2 rectal cancers, compared with T3 tumors [14-18]. Therefore, most patients with clinically staged T1-2 node-negative rectal cancer will undergo primary resection, and adjuvant treatment will be determined according to the pathological stage. For the small subset of patients with pT1-2 node-positive rectal cancer, the optimal combination of surgery, chemotherapy and radiotherapy is still controversial. Based on outcomes data, adjuvant chemotherapy is recommended for all patients with node-positive rectal cancer [19,20]. Consistent with this recommendation, in our series, the 5-year DM rate for patients with pT1-2 node-positive rectal cancers who received adjuvant chemotherapy was 25.8%, which was obviously lower than patients without any adjuvant therapy (5-year DM rate, 49.5%).

We sought to determine whether or not adjuvant radiotherapy improves local control following an optimal surgery. An optimal TME for T1-2 tumors can successfully resect the primary tumor, dissect the perirectal fascia and clear any involved lymph nodes [21-23]. In our series, local control was similar between patients who did or did not receive adjuvant radiotherapy. By analyzing the patients within more detailed TN stage subsets, we found that the 5-year LR rate was 26.4% for patients with pT2N2 rectal cancer, which was almost five times that with pT1N1 tumors (5.3%). Of all the 46 patients with pT1N1 tumors, there were only 2 cases of local recurrence. Although it is difficult to provide a persuasive conclusion, the optimal local control suggested obviating radiotherapy may be reasonable in pT1N1 patients. However, for 42 patients with pT2N2 tumors, although in a small number of cases, local recurrence rate was greatly reduced by adjuvant CRT, which suggested the benefit of adjuvant CRT in these patients. Therefore, our data indicate that adjuvant CRT is warranted for patients with pT2N2 rectal cancer.

Unlike with colon cancer, prevention of local recurrence is a major concern in treatment decision making for rectal cancer. It is difficult to define the risk of local recurrence according to the TNM stage. In a pooled analysis of three North American randomized phase III rectal adjuvant trials, patients were categorized into three groups with distinct DFS and OS [4,24-26]. Because all patients received postoperative radiotherapy, it was impossible to compare local control with or without radiotherapy. Only a 2% difference in local control was observed for patients with pT1-2 N1 tumors vs. pT1-2 N2 tumors. Data from an additional two randomized phase III North American rectal cancer adjuvant studies [26,27] was added to allow for a comparison of outcomes following multiple treatment modalities (surgery alone, surgery + radiotherapy, surgery + CRT and surgery + CT) [28]. In this analysis, patients with pT1-2 N1 and pT1-2 N2 tumors experienced similar improvements in local control with any adjuvant treatment. Patients with pT1 and pT2 tumors were combined when comparing outcomes. By contrast, our study showed that distinct LR and DM rates exist among patients with pT1 and pT2 node-positive rectal cancers. To improve local control and minimize potential harm from overtreatment, adjuvant therapy should be individualized for these subsets of patients.

We were unable to determine whether adjuvant CRT is warranted in patients with Intermediate-risk (pT1N2 and pT2N1) rectal cancer. Although it might be possible to improve the 5-year LR rate to less than 10%, we believe that over 35% 5-year DM rate should prioritize combination chemotherapy in this patient subgroup. It is important for physicians to assess the tolerability of adjuvant CT only or CRT in advance. For patients of intermediate risk, it may be beneficial to consider adding radiotherapy to adjuvant treatment if combined chemotherapy was well tolerated.

T1-2 rectal cancer is generally considered to be a local disease that is unlikely to result in regional or distant metastasis. The intrinsic cause of metastasis in T1-2 rectal cancer is unknown. The tumor invasion depth (T2 vs. T1), or submucosal invasion in T1 patients (sm1-3) may not correlate with lymph node metastasis [15,29]. Of note, 53% of patients in our study had T1N2 disease. Some studies have found that markers of aggressiveness, vascular invasion or tumor grade, associate with early lymph node metastasis [5,30,31]. Further study of the biological features of these cases may improve our understanding of invasion by rectal cancer cells.

## Conclusions

In our study, we provide the detailed outcomes and preliminary survival analysis in a relatively infrequent subset of rectal cancer. Patients with T1-2 node-positive rectal cancer can be classified into three risk subsets based on local control rates. Great treatment disparities exist in pT1-2 node positive rectal cancer. Postoperative treatment needs to be individualized for these patients. This retrospective analysis suggests adjuvant CRT may be warranted in patients with pT2N2 rectal cancer, while the benefit in patients with pT1N1 rectal cancer was still undetermined.

## Competing interests

There are no financial competing interests to declare in relation to this manuscript.

## Authors’ contributions

Study conception and design: JP, XL, SC. Acquisition of data: DS, XL, HW. Analysis and interpretation of data: YD, JP. Writing manuscript: JP, YD, HW. All authors read and approved the final manuscript.
